# Evaluation of newly copolymers and their montmorillonite nanocomposite as cold flow improver for petroleum lubricating oil

**DOI:** 10.1038/s41598-023-41802-1

**Published:** 2023-09-11

**Authors:** Alshaimaa H. El-Bahnasawi, Abeer A. El-Segaey, Salwa A. H. Albohy, Olfat E. El-Azabawy, Enas I. Arafa, Nagda G. El-Koly, Hussin I. Al-Shafey

**Affiliations:** 1 Petroleum Applications Department, Egyptian Petroleum Research Institute, Nasr City, Cairo Egypt; 2 Chemistry Department, Faculty of Science, Al-Azhar University, Nasr City, Cairo Egypt

**Keywords:** Chemistry, Energy science and technology, Nanoscience and technology

## Abstract

The great demand on the energy makes the attention toward modifying lubricating oil. This work tends to prepare the following copolymers; octadecylmethacrylate-co-dodecene (CP_1_) and octadecylmethacrylate-co-hexadecene (CP_2_) by free radical solution polymerization using laboratory prepared octadecylmethacrylate monomer with either 1-dodecene or 1-hexadecene. The same monomers also used to prepare their polymers nanocomposite (NP_1_, NP_2_) with 1% of nanomontmorolonite by emulsion polymerization. The structures of the prepared polymers and their nanocomposite were elucidated by FTIR, ^1^HNMR, TGA, DSC, TEM and DLS. These polymers were used as pour point depressant, flow improver and viscosity modifier and showed high efficiency. After comparison of the data of the polymers and their nanocomposite, the nanocomposite give the best results where the pour point decreased from 0 °C to − 18, − 27, − 24 and − 33 °C for CP_1_, CP_2_, NP_1_ and NP_2_ respectively at the optimum concentration 10,000 ppm. On the other hand the viscosity index increased from 86.57 to 93.25, 92.41, 94.17 and 93.103 for CP_1_, CP_2_, NP_1_ and NP_2_ respectively, the apparent viscosity increased from 55.863 to 69.31, 119.41, 111.28, and 166.89 cP also the yield stress increased from 652.19 to 1076.3, 1074 and 1480 D/cm^2^ for CP_1_, CP_2_, NP_1_ and NP_2_ respectively.

## Introduction

In the automobile sector, lubricating oil is a substance that is frequently used^1^. Lube oil forms a barrier layer serving as protection for machinery parts, and so reduces the friction that occurs during the turbo-chemical process^2^. Besides that, the engine efficiency is increased as a result, prolongs their life as well as economizes energy but it contains high wax content. Thermal, mechanical, or chemical management methods are frequently implemented as part of flow assurance procedures for the prevention, mitigation, and correction of production issues brought on by paraffin wax^3,4^. According to the depression in the temperature, wax deposition causes serious issues for ensuring the flow of undersea pipelines^5,6^. The investigation of wax mitigation methods, such as hot water or oil or by adding pour-point depressants, wax crystal modifiers^9^, also by mechanical–biological methods, has taken up a lot of time and effort in recent years^7,8^. The chemical treatment approach has been employed among these treatment methods to effectively reduce the deposition of the wax even at the lowest dose and less expenses than these other methods^9^. The reason for the poor fluidity is the development of a net-like or cage-like structure brought on by the precipitation of wax that typically categorized as macro-crystalline or microcrystalline waxes^10–13^.

Polymers that used to make the oil flow at lower temperatures without creating wax and also keep the oil able to be pumpable under these chilly conditions (flowable) that called pour point depressants (PPD) were used for treating the paraffinic engine oils^14^. These PPDs may contain the green, natural polymers^15,16^ and methacrylate polymers.

Methacrylate polymers were applied as pour-point depressants and viscosity modifiers depends on the length of the alkyl chain also on the oil type^17,18^. In this context, polymethacrylates (PMAs) having long n-paraffins as pendant chains (ranging from 16 to 20 carbon atoms) are expected to be more efficient. Mixed PMA esters having 1–22 pendant C-atoms were reported as good PPD with regard to poor PPD properties of homopolymers and single ester^19^. It was found that methacrylates (contain at least 14-C atoms) must be a component of these additives in order to have depressant characteristics^20^. However, application of nanomaterial as additives opens new horizons in the lubricant industry^21^. Numerous studies have been conducted recently to assess the potential for a variety of inorganic nanoparticles to act as additive agents for lubricating oil^22^ due to the distinctive advantages of the particular lamellar structure and the effective dispersion of nanoparticles. Due to their outstanding capabilities, nanoparticles are increasingly being used as lubricant additives^23,24^ where they are able to enter the friction area and fill the micro-roughness because of their small diameters (2–100 nm)^25^. This process prevents the friction surface from coming into direct touch with one another^26^. The high surface energy of the nanoparticles helps to easily form a protective film on the friction surface also exhibit self-repairing effects on obsolete surfaces^27–29^.

This work targets preparation of octadecyl methacrylate (ODMA) copolymers with either 1-dodecene (DD) or 1-hexadecene (HD) (ODMA-DD and ODMA-HD) via free radical polymerization. Additionally, nanocomposites of both ODMA-DD and ODMA-HD by emulsion polymerization using 1% NMMT (nano-montmorillonite), the new synthesized copolymers (CPs) and their nanocomposites were used as novel flow improver and pour point depressant for local lubricating oil. Comparing the copolymers and their nanocomposites, it was found that the nanocomposites give better efficiency as cold flow improver (FI) for lube oil. The effective properties of the recently created FIs were proven by the detection of the pour point temperature (PPT), viscosity-index (VI) and rheological-properties (RP). Moreover various analyses were performed to prove their chemical and physical properties including FTIR, ^1^HNMR, TEM, DLS and DSC.

## Experimental

### Materials

This report is conducted by many chemicals from Sigma Aldrich Chemical Co.; stearyl alcohol (> 99), methacrylic acid (99%), 1-dodecene, 1-hexadecene, nano montmorillonite (NMMT), hydroquinone, p-toluenesulfonic acid (P-TSA), benzoyl peroxide (initiator) and sodium salt of dioctyl-sulfosuccinate (surfactant). Used solvents are from Edwic Co including toluene, xylene, hexane, methanol and sodium carbonate. The substances were analytical grade reagents except benzoyl peroxide (chemical grade). Lubricating mineral oil was from Co-operation Company, Egypt, Mobil DTE Oil purchased from Watanya gas station, Egypt.

### Synthesis of octadecyl methacrylate

**Scheme 1:**
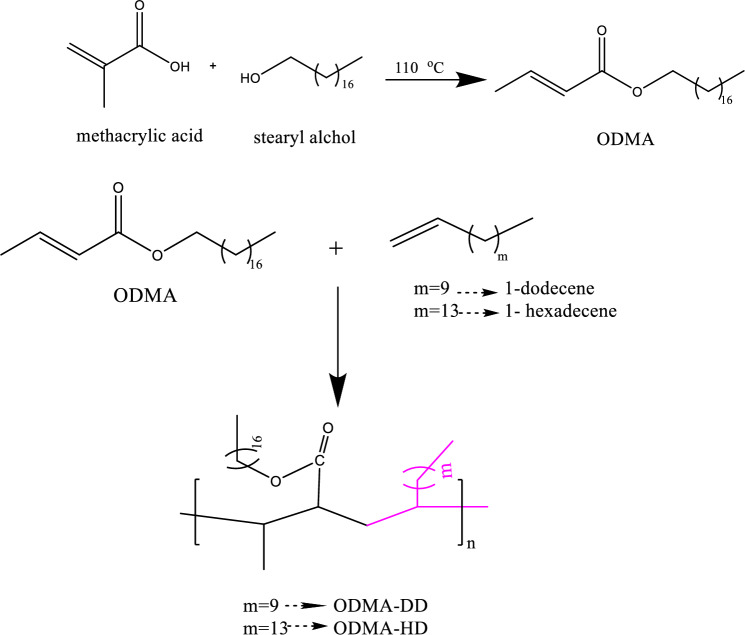
The reactions steps involved in preparing esters and copolymers (CPs).

Octadecyl methacrylate (ODMA) was synthesized through esterification of methacrylic acid with stearyl alcohol in a 1:1.1 molar ratio with p-PTSA, and hydroquinone in 50 mL toluene. A three-neck flask with Dean-Stark apparatus connected to a condenser was used for this reaction; water was collected in the Dean-Stark^30,31^. The reaction mixture was gradually warmed up to 110 °C from room temperature. Monitoring the quantity of water released during the reaction allowed us to determine how far the reaction had progressed. The prepared ester of stearyl methacrylate was washed repeatedly with Na_2_CO_3_ solution (5%) until became clear then rinsed with distilled water and let to dry under vacuum. The reaction illustrated in the Scheme 1.

### Synthesis of copolymers

The polymerization reaction was done by the reaction of the prepared octadecyl methacrylate (ODMA) with either 1-dodecene (DD) or 1-hexadecene (HD) with molar ratio 1:1for preparation of poly (ODMA-DD) and poly (ODMA-HD) respectively. The initiator and toluene were also added to the system. For 6 h, a temperature of 95 °C was maintained for the process. After that, the reaction was stopped by dropping the mixture to the methanol while stirring, a precipitate then formed. By repeatedly precipitating the copolymers from their hexane solution with methanol, followed by vacuum-assisted drying at 70 °C. The reaction illustrated in the Scheme 1.

### Synthesis of polymeric nanocomposites

**Scheme 2:**
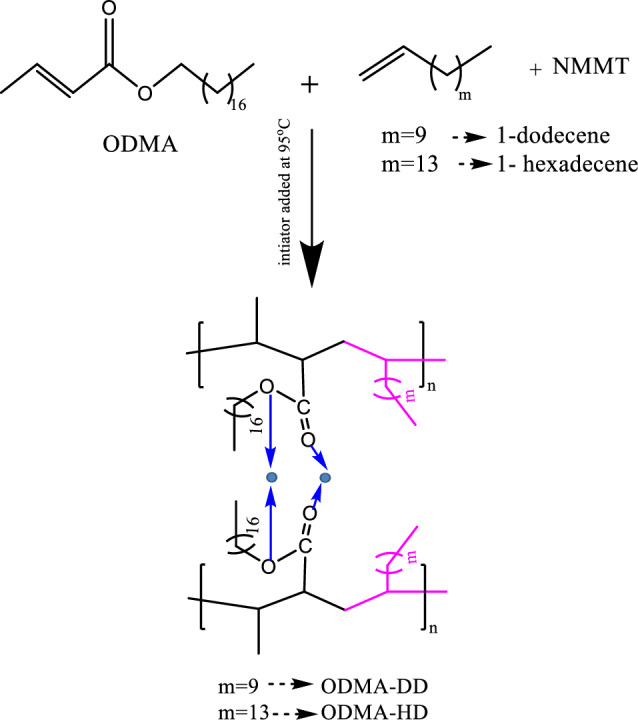
The reaction steps for synthesis of copolymer and its nanocomposites (NPs).

Copolymers’ nanocomposites (ODMA-DD/NMMT, ODMA-HD/NMMT) were synthesized by emulsion polymerization reaction of the corresponding monomers and NMMT. To begin, 1% of NMMT in dist. water was let to stirring overnight then added to the sonicatted mixture of the monomers in toluene also the toluene solution of surfactant was added. All the content was subjected to N_2_ gas and the initiator was added to start the polymerization when the temperature reaches 95 °C^32^. A mixing speed of 450 rpm was used during the 24-h polymerization process. The mixture was let to cool at room temperature then washed with dist. water for elimination of the used surfactant. The reaction illustrated in the Scheme 2.

### Measurements

In order to get the Fourier Transform Infrared Spectrometer (FTIR) spectra of the produced polymers and their nanocomposite in the 4000–400 cm^−1^ wavenumber range, FTIR (USA) was applied by using potassium bromide (KBr).

Proton nuclear magnetic resonance spectroscope (^1^H NMR) was used to prove the chemical structure of the prepared polymers and their nanocomposite by applied the Avance III with 500 MHz (Switzerland) also deuterated chloroform was employed as the solvent, and tetramethylsilane served as the internal standard.

Dynamic light scattering (DLS) that used to measure the size was operated by a Brookhaven + 90 model with size/zeta potential analyzer (USA).

HR-TEM (JEM2100LaB6 at 200 kV0.14 nm resolution, Japan) was used to accurately measure the nanoparticles' sizes. An ultrasonicator was used to disperse the polymers in ethanol before it was placed onto a copper grid that had been covered in carbon. Leica Ultracut UCT is used to perform ultra-cryomicrotomy on the nanocomposite samples before TEM examination (Leica Microsystems GmdH, Vienna, Austria)^33^. Cryosections with a thickness of between 100 and 150 nm were made at a temperature of 150 °C using freshly sharpened glassy knives with 45 cutting edges. The sections were individually collected and then directly applied on a copper dried grid with a mesh size of 300.

TGA and DSC were used to study the thermal stability of the prepared copolymers by the SHI- MADZU DTG-60 type of thermo gravimetric analyzer using an alumina crucible in the air with heating-rate 10 °C/min, while the test temperature starts from 0 till reach 550 °C.

GPC method (HLC-8320 Gel Permeation Chromatography) can be used to measure polydispersity and molecular weights.

In order to determine the wax crystal shape of lubricating oil samples, Lecia DM4 polarizied microscope (Germany) was used. At a 100 × magnification, the photos were captured at roughly 0 °C.

Using the ASTM D-97 Standard technique, the pour points of both treated and untreated lubricating oils were measured^34^.

The rheological properties were studied by using Anton-Paar MCR302 rheometer at different temperature, 0, 15, 25 and 35 °C and shear rate 5 s^−1^.

The ASTM D2270 standard procedure was used to calculate the viscosities indices of the lubricating oil prior and following the treatment of 10,000 ppm of the additives^35^. In this regard, the kinematic viscosities at 40 °C and 100 °C were detected^36^.

## Results and discussion

### FTIR spectroscopy

**Figure 1:**
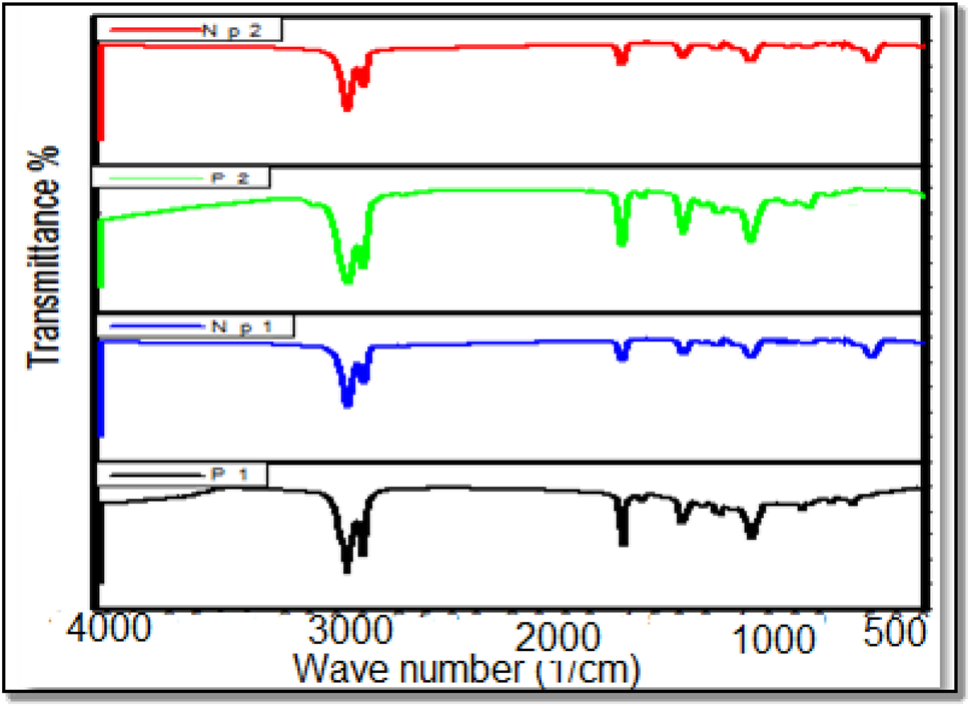
FT-IR spectra of polymers and nanocomposite.

The FTIR spectra of (ODMA-DD), (ODMA-HD), (ODMA-DD/NMMT) and (ODMA-HD/NMMT) were shown in Fig. 1. The following peaks are obtained: 2927 cm^−1^ and 2851 cm^−1^ are corresponded to C–H, 1732 cm^−1^ is attributed to C=O, 1435 and 1237 cm^−1^ assigned to C–O and C–C. All spectra did not show any peak at 1639 cm^−1^, that demonstrates the synthesis of polymers. For nanocomposites’ charts extra peaks are formed viz. 1152 cm^−1^ attributed to Si–O–Si, 625 cm^−1^ assigned to SiO_2_ that prove the compose of the polymer nanocomposites^37,38^.

### ^1^H NMR spectra of CPs and their NPs

**Figure 2:**
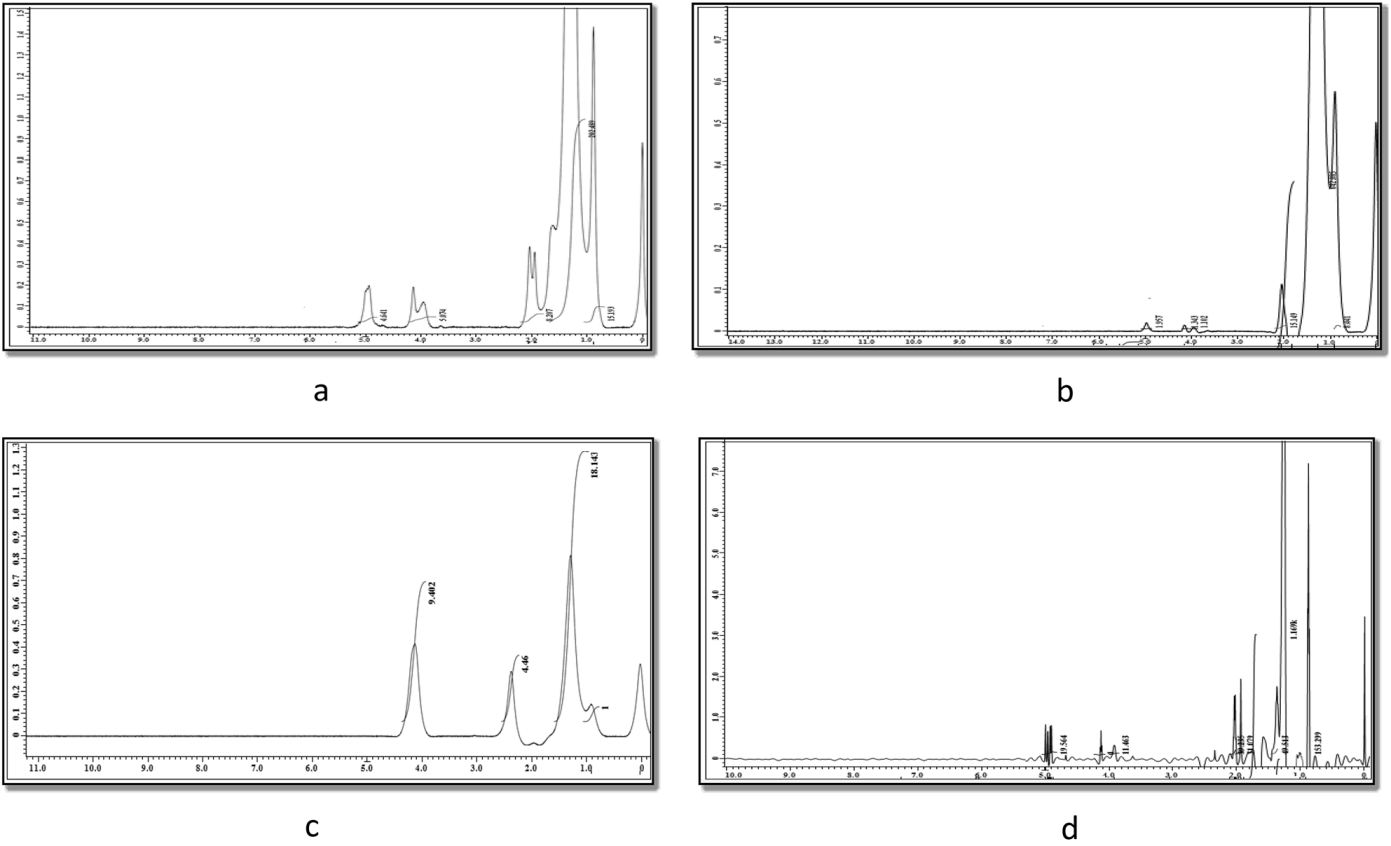
(**a**) ^1^HNMR of CP_1_. (**b**) ^1^HNMR of CP_2_. (**c**) ^1^HNMR of NP_1_. (**d**) ^1^HNMR of NP_2_.

The ^1^HNMR of the copolymers (CPs) and their nanocomposites (NPs) were illustrated at Fig. 2a–d. The resonance at 0.9 ppm, 1.2 ppm and 1.6 ppm are attributed CH_3_, CH_2_ and CH, respectively. The resonance in the range of 1.96–2.37 ppm assigned to COCH whereas that appeared, at 3.6 ppm is ascribed to COCH_2_ and that at 4.13–4.3 ppm is to attributed OCH.

### TGA and DSC analysis

**Figure 3:**
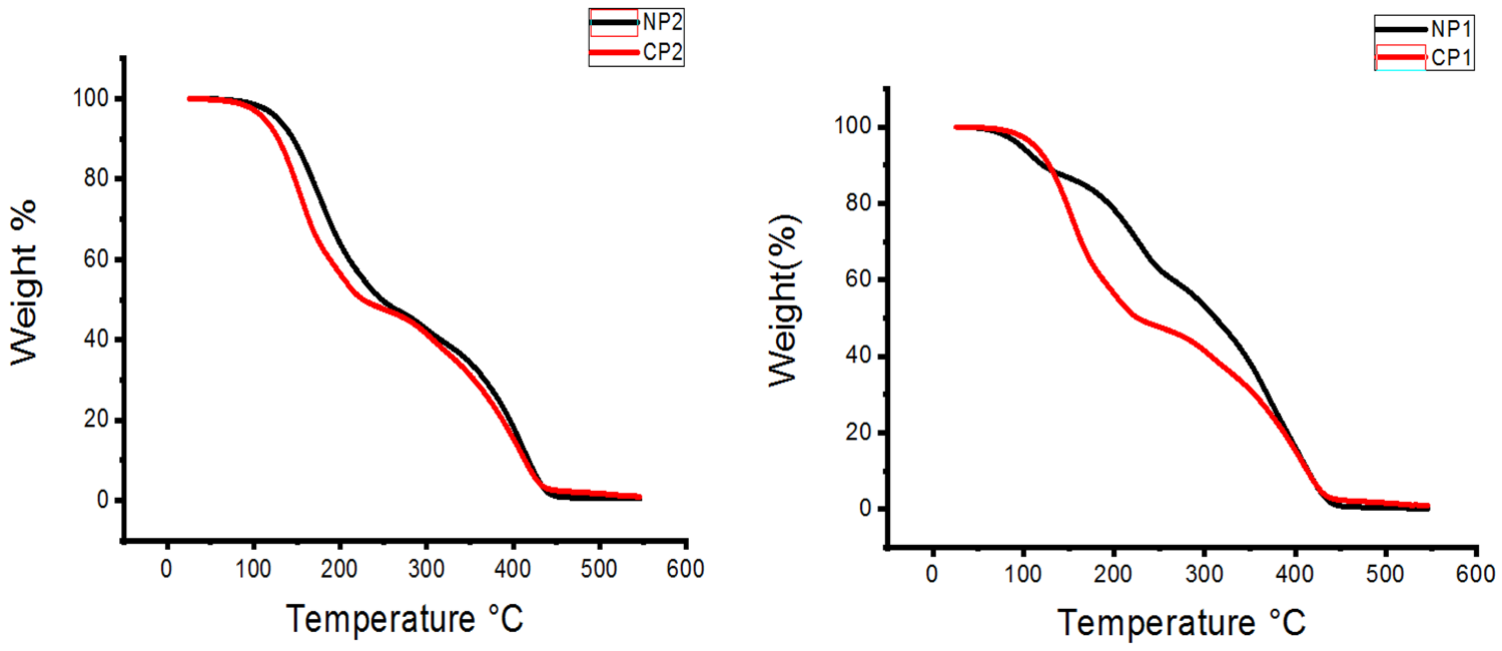
TGA analysis of polymers and its nanocomposites.

The thermal behavior of the copolymers and their nanocomposite were studied through the Thermo gravimetric analysis and were illustrated in Fig. 3. There are three stages of decomposition in the samples' estimated thermo-gram values; For the first stage a weight loss of 20% was calculated for CPs at a temperature from 20 to 100 °C to the loss of water, but at NPs the loss occurs at temp from 220 to 250 °C (40% loss). Furthermore, the following stage was predicted to occur between 100 and 200 °C for CPs (30% loss) but occur between 250 and 400 °C for NPs (30% loss).The final stage, CPs was found at temp. 400 and 600 °C (50% loss) but NPs at this temperature give (0% loss).

These results could be attributed to the thermal stability of both copolymers and their nanocomposite. Also the Nps show high stability than that of CPs this also could be attributed to the presence of the nano particles inside the matrix of the polymer epically NMMT show high resistance to the thermal decomposition^39,40^.

**Figure 4:**
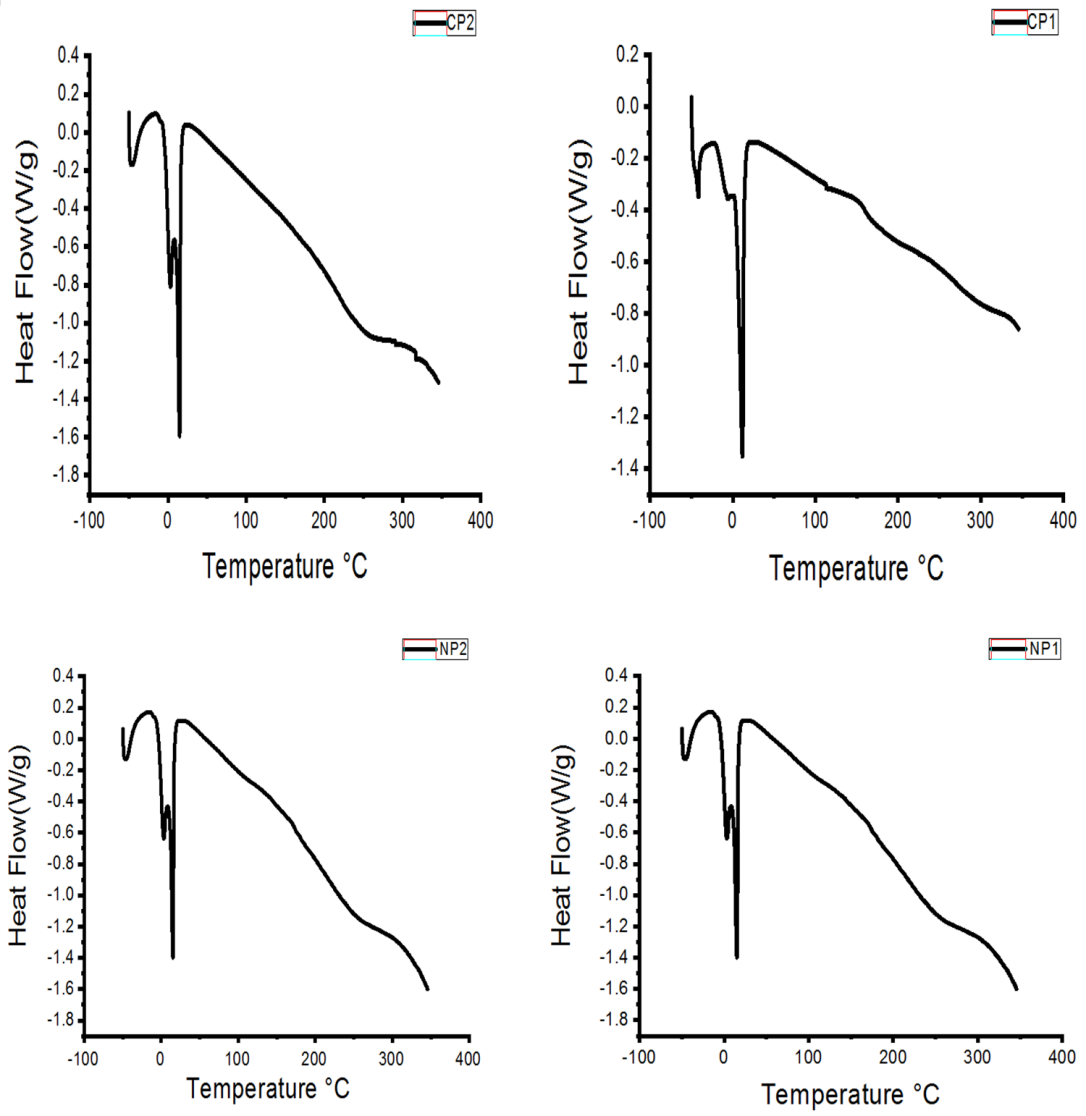
DSC analysis of two polymers and its nanocomposites.

Differential scanning calorimetry (DSC) is used to predict if the compound endothermic or exothermic through measurement of the transformation-temperatures and enthalpy with respect to temperature so the sample was maintained at the same temperature of the reference the record the heat-flow. DSC curves of the prepared polymers and their nanocomposite were shown in Fig. 4. The graphs display a number of endothermic peaks between 75 and 500 °C referring to the temperature of melting and degradation of the polymers. According to the curves, it is obvious that addition of nanoparticles increases the thermal stability of the polymers this is due to interaction between the polymer particles and NMMT that coagulated at the polymer surface.

### DLS and HR-TEM

**Figure 5:**
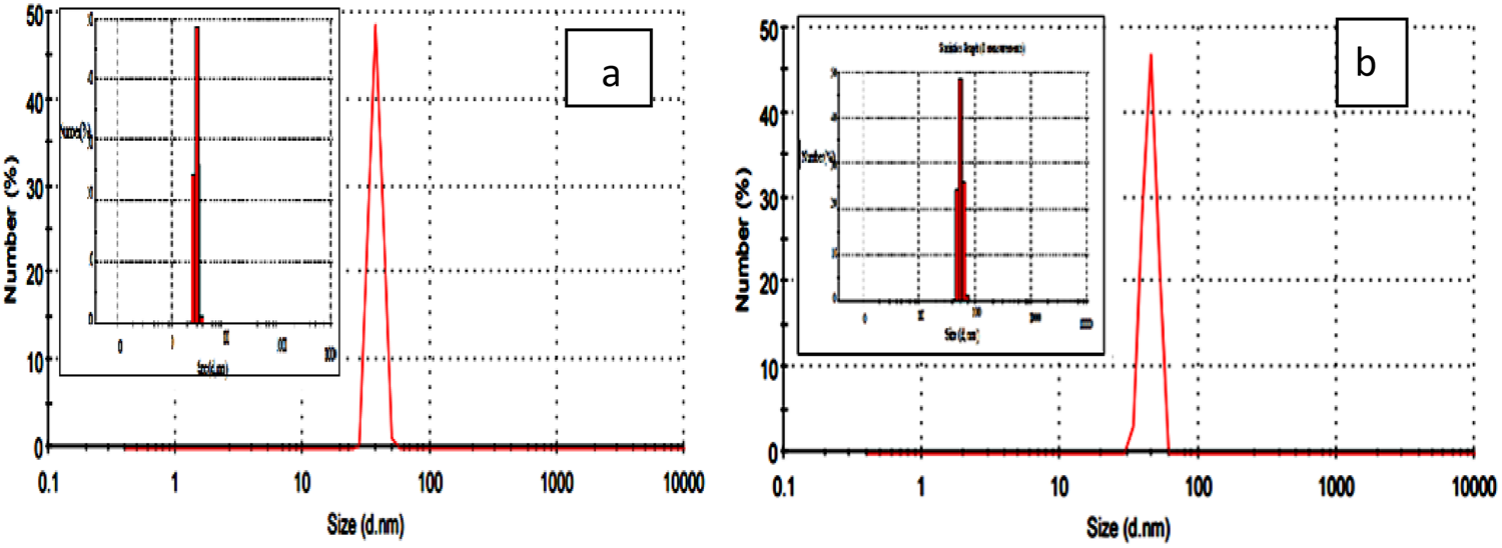
DLS photos of (**a**) NP_1_ and (**b**) NP_2_.

Dynamic light scattering (DLS) is measured to understand the distribution of the polymer nanocomposite's particle size in its prepared form. Figure 5a,b reveals that the particle sizes in the size distribution profiles are 78 and 83 nm for NP1 and NP2, respectively, indicating that the nanocomposite was successfully formed.

**Figure 6:**
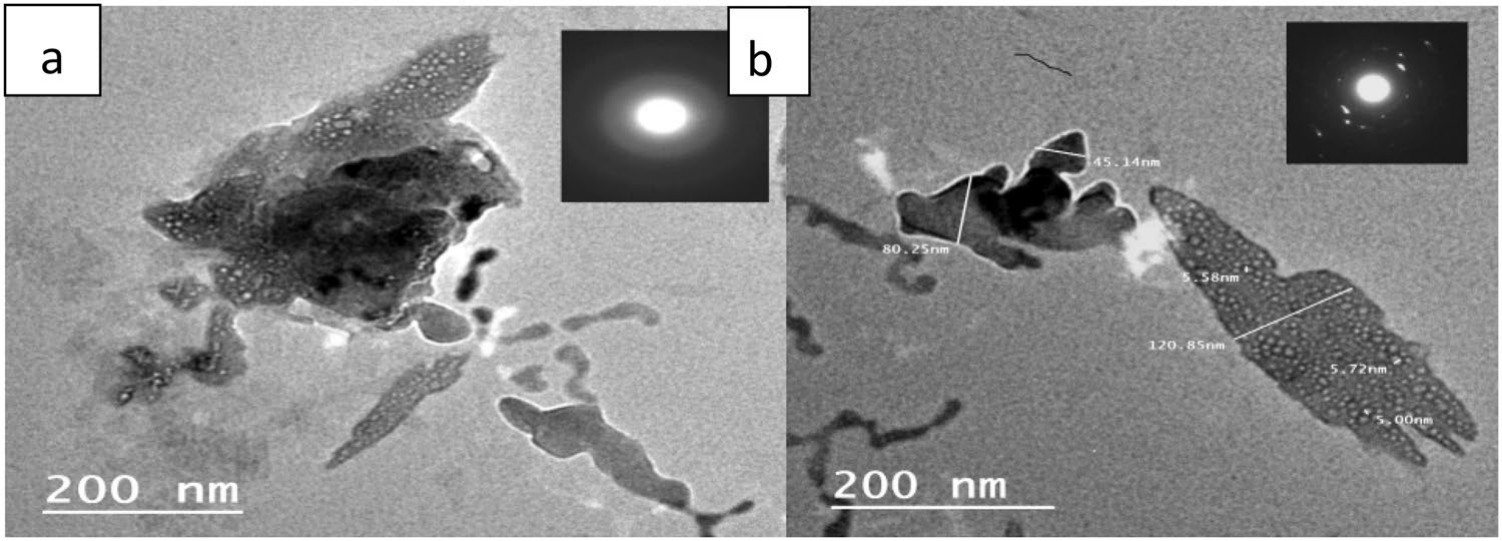
HR-TEM images of (**a**) NP_1_ and (**b**) NP_2_.

TEM images were illustrated at Fig. 6a,b. These images show the successful preparation of the copolymer nanocomposite, where the NNMT (white dots) is distributes through the copolymers (black part).

### Evaluation the CPs and NPs as PPDs

**Figure 7:**
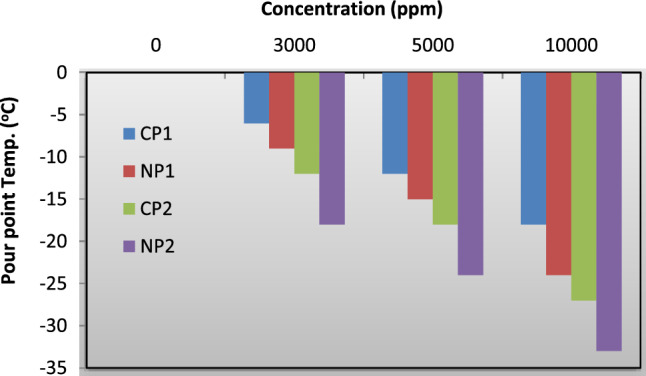
Relation between pour-point temperature and concentration.

The temperature that stops the oil from flowing when pulled downward by gravity is known as the oil pour point^41–43^. Machine startup in cold climates is made challenging or impossible by oil with a high pour point. Paraffin wax, which has a tendency to crystalize, is what gives cold oil its hardness^44,45^. Addition of the Pour-point depressant decreases the ability of the wax to be crystalized and also lowing the temperature of the pour-point. Lubricating oils' storage and functionality can be severely hindered by waxes^46,47,54^. The results of the pour-point temperatures at different concentrations were tabulated at Table 1 then drawn in Fig. 7. The effectiveness of these prepared additives is increased by increasing their doses, which caused the pour points for CP_1_ and CP_2_ to drop from 0 to − 18 and − 27 °C, respectively. The sharp decrease occurs at the treatment with polymer nanocomposite where the PPT decreased from 0 °C until − 24 and − 33 °C for NP_1_ and NP_2_ respectively**.**

**Table 1:** Pour-point temperature of the untreated lubricating oil (0 °C) and that treated with different concentration of CP_1_, CP_2_, NP_1_ and NP_2_ and their average molecular weight.

Sample	Molecular weight × 10^3^	PPT (°C)		
		3000 ppm	5000 ppm	10,000 ppm
CP_1_	70.35	− 6	− 12	− 18
NP_2_	76.1	− 9	− 15	− 24
CP_2_	103.8	− 12	− 18	− 27
NP_2_	119.45	− 18	− 24	− 33

### Determination of viscosity index

In order to assess how effective the synthesized polymeric additives are as viscosity modifiers, viscosity index of the polymeric additives was measured for the lubricating oil^53–55^. At 40 and 100 °C, the kinematic viscosity is measured for the lubricating oil before and after treatment with 10,000 ppm of polymers and composites, the data are presented in Table 2. Adding the additives to lubricating oil enhanced the viscosity index (VI) and by comparison between the results of the CPs and NPs show that the NPs is the best as flow improver, where VI was calculated to be 86.57 for a blank sample of lube oil, on the other hand, treatment with either CP_1_ or CP_2_ the VI becomes 93.25 and 92.41, respectively but in case of treating with NP_1_ and NP_2_ the VI becomes 94.17, 93.103 respectively. All these data referring to these copolymers and their composite could be used as flow improver especially that the NPS (the best results).

**Table 2:** The viscosity index of the pure lube oil and that treated with 10,000 ppm of CPs and NPs.

Sample	Kinamatic viscosity, cSt ASTM D-445		Viscosity index
	40 °C	100 °C	
Blank	56.86	7.348	86.57
CP_1_	58.24	7.659	93.25
NP_1_	63.40	8.104	94.17
CP_2_	60.12	7.786	92.41
NP_2_	56.18	8.381	93.103

### Rheological properties

**Figure 8:**
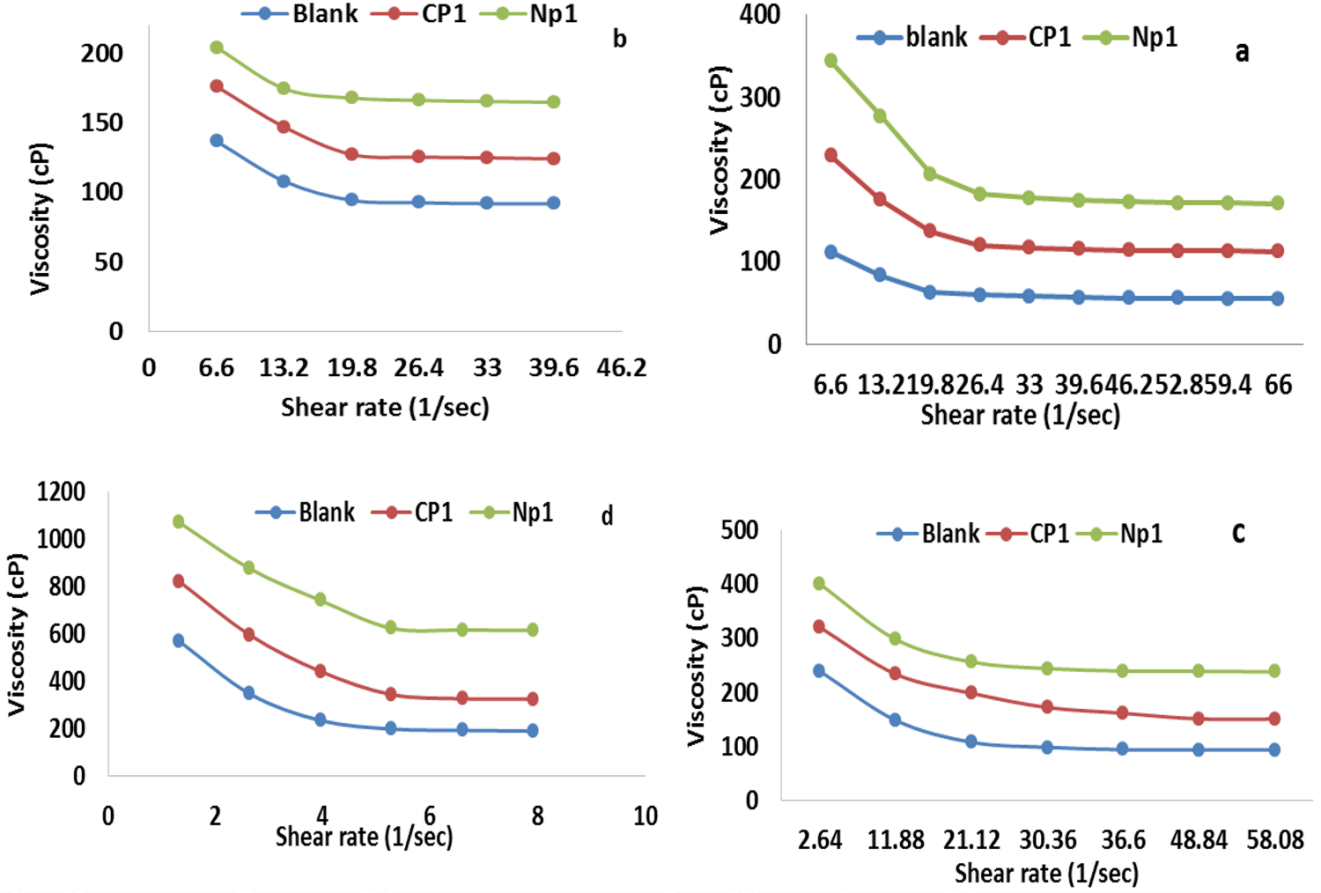
Relationship between apparent viscosity and shear rate for lubricant oils that have been treated and those that haven't with 10,000 ppm from CP_1_ and NP_1_ at temperatures (**a**) 35 °C, (**b**) 25 °C, (**c**) 15 °C, and (**d**) 0 °C.

**Figure 9:**
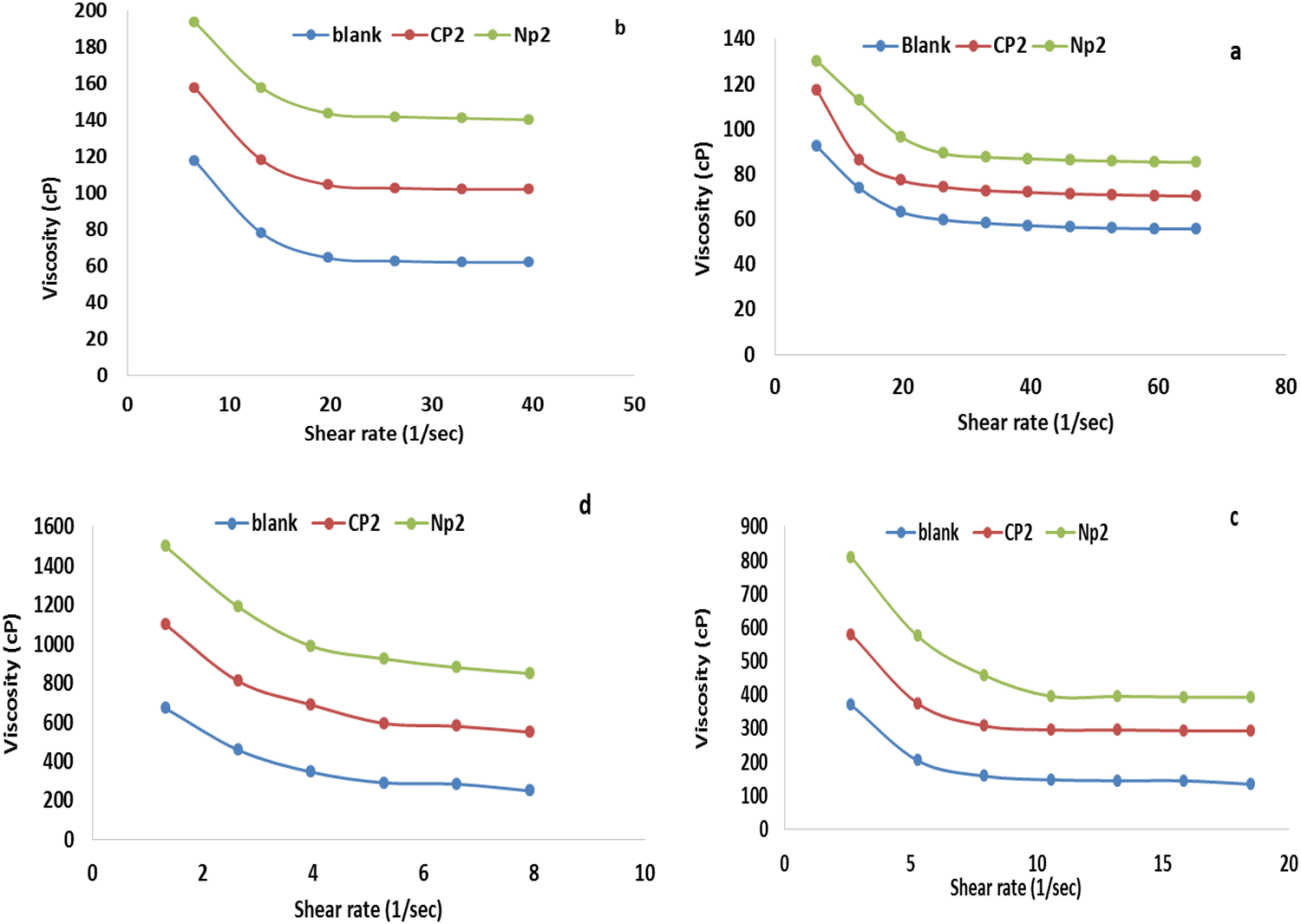
Relationship between apparent viscosity and shear rate for lubricant oils that have been treated and those that haven't with 10,000 ppm from CP_2_ and NP_2_ at temperatures (**a**) 35 °C, (**b**) 25 °C, (**c**) 15 °C, and (**d**) 0 °C.

**Figure 10:**
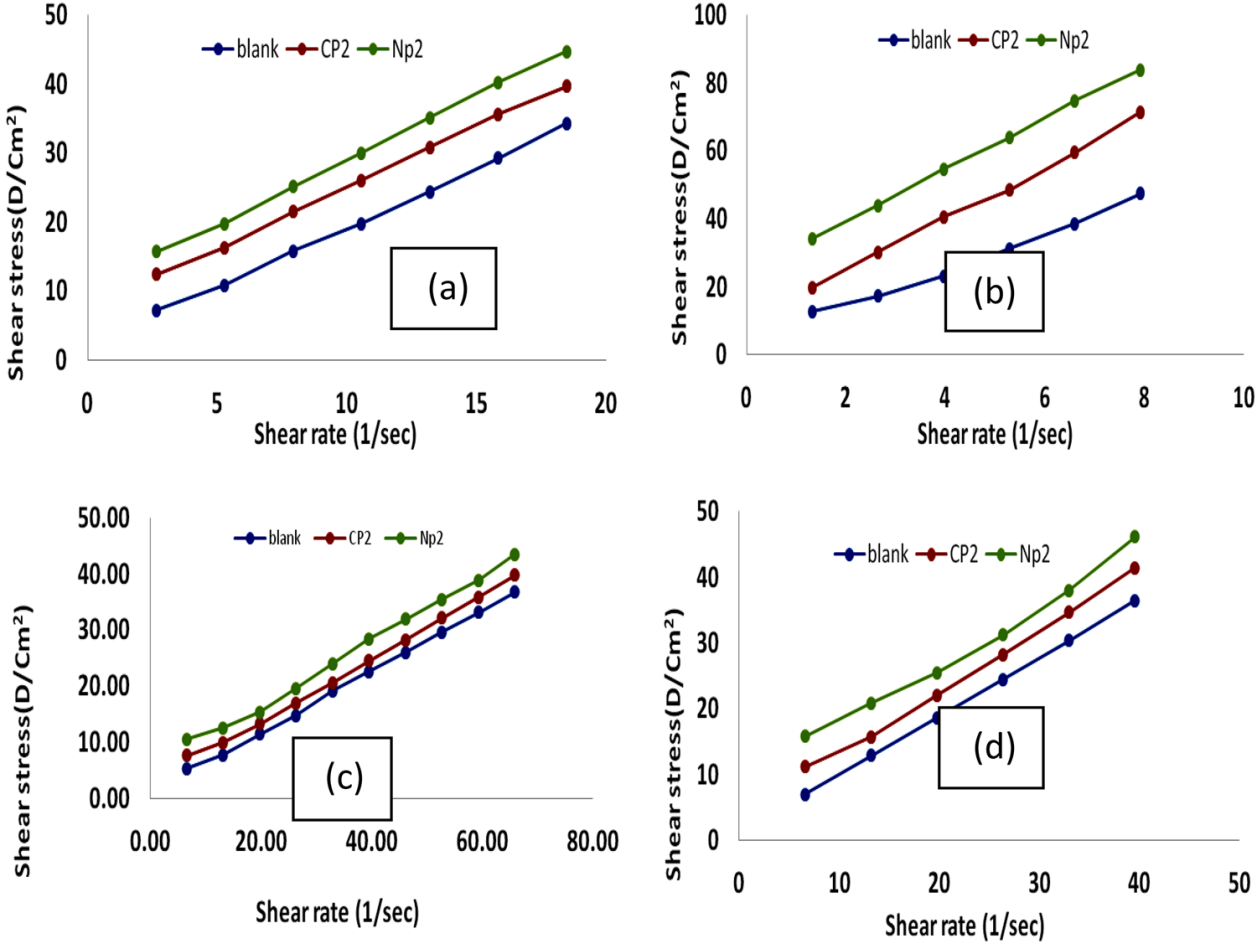
Relationship between shear rate and shear stress using 10,000 ppm of treated and untreated lubricating oil from CP_1_ and NP_1_ at temperatures (**a**) 35 °C, (**b**) 25 °C, (**c**) 15 °C, and (**d**) 0 °C.

**Figure 11:**
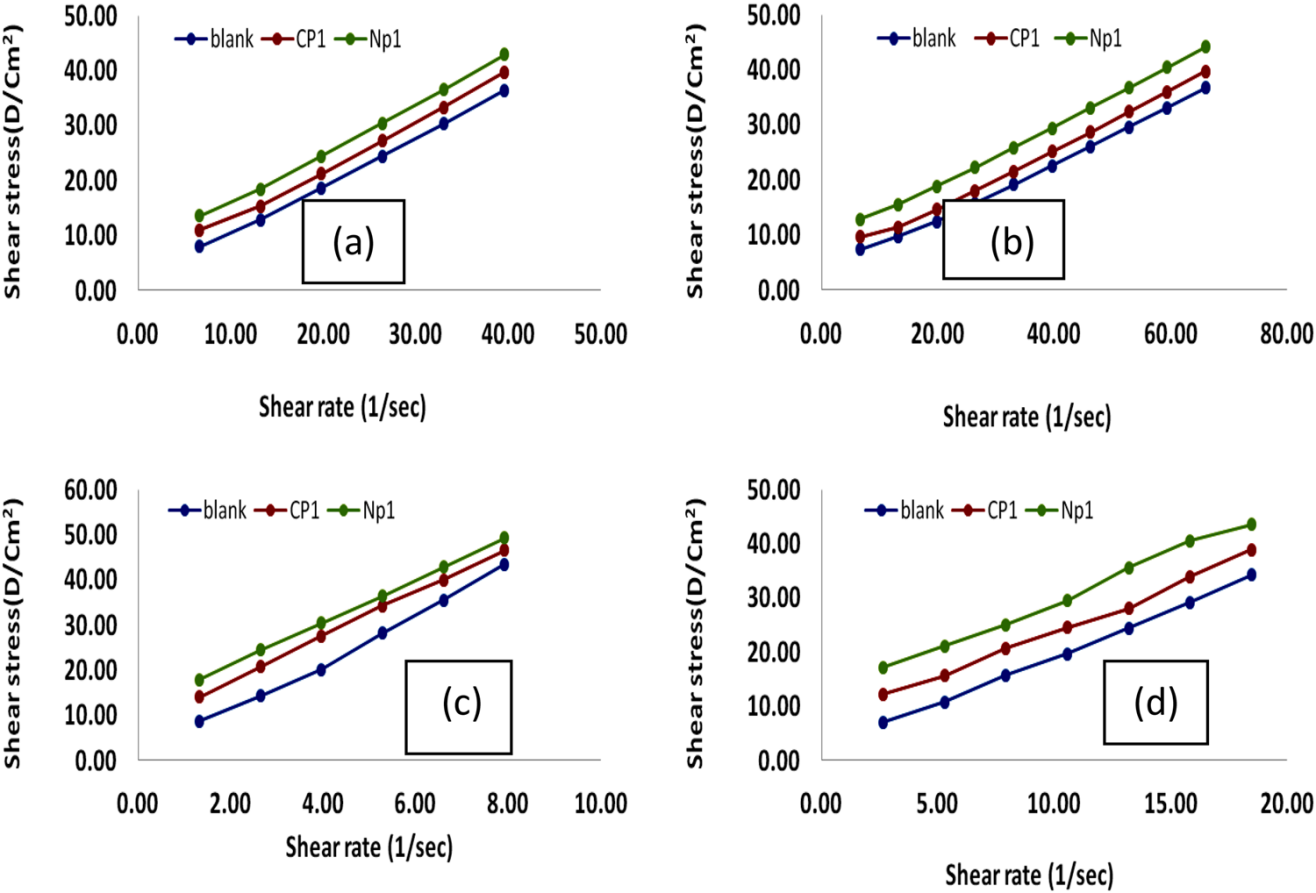
Relationship between shear rate and shear stress using 10,000 ppm of treated and untreated lubricating oil from CP_2_ and NP_2_ at temperatures (**a**) 35 °C, (**b**) 25 °C, (**c**) 15 °C, and (**d**) 0 °C.

Dynamic viscosity measurements were conducted at 0, 15, 25, and 35 °C to assess the rheological behavior of the lube oil that had not been treated and that had been treated with CP and PNs additions in the most effective concentration (10,000 ppm). The viscosity-shear rate curve was plotted as illustrated in Figs. 8, 9 and shear stress versus shear rate is illustrated in Figs. 10 and 11.

From the Figs. 8 and 9 can note that the viscosity decrease with increasing the shear rate also decrease with increasing the temp. But by comparison between the viscosity of the lubricating oil that treated with the additives with that pure lubricating oil could note that the viscosity increase with the adding the additives. This phenomenon can be explained through raising the shear-rate, which reduces the waxy agglomerates and causes part of the continuous phase that had been immobilized within the agglomerates to be released^48,49^. At all studied samples, particularly at high temperatures, the viscosity reduces roughly linearly with the increase in shear rate^50^. In lubricating oils, wax crystals tend to aggregate, but this is prevented by the strong polarity of oxygen in the ester group along the polymer chain^46^. The estimated experimental data of shear rate and shear stress measurements that shown in Figs. 10 and 11 using the Bingham plastic model, the infinite viscosity of the shear-rate is known as apparent viscosity^51,52,55,56^. Table 3 lists the results of both of the apparent viscosity and the yield stress that obtained from the curves of shear stress at various temperatures. Overall, as shown at the table, Bingham model was consistently demonstrated a typical increase in apparent viscosity and yield stress values with lowering temperatures for both untreated and treated lubricating oil samples. It demonstrates that the apparent viscosity and yield stress of treated lube oil with CP_1_, CP_2_, NP_1_ and NP_2_ give higher results than that pure lubricating oil, where the apparent viscosity rising from 55.863 to 69.31, 119.41, 111.28, and 166.89 cP and the yield stress rising from 652.19 to 1076.3, 1074, and 1480 for CP_1_, CP_2_, NP_1_ and NP_2_, respectively.

**Table 3:** The apparent viscosity and yield stress values.

Sample	Temp., °C	Apparent viscosity, cP	Yield value, D/cm^2^
Blank	0	55.863	652.19
	15	11.83	306.08
	25	2.3306	107.37
	35	1.2607	79.523
CP_1_	0	69.31	816.61
	15	4.0851	281.37
	25	3.5377	147.32
	35	2.7439	97.871
CP_2_	0	119.41	1076.3
	15	6.0107	346.03
	25	5.8931	196.48
	35	3.8539	99.594
NP_1_	0	111.28	1074
	15	23.602	496.35
	25	3.7292	170.37
	35	1.5794	98.257
NP_2_	0	166.89	1480
	15	32.476	727.78
	25	5.0832	185.03
	35	1.9158	116.43

### POM analysis

**Figure 12:**
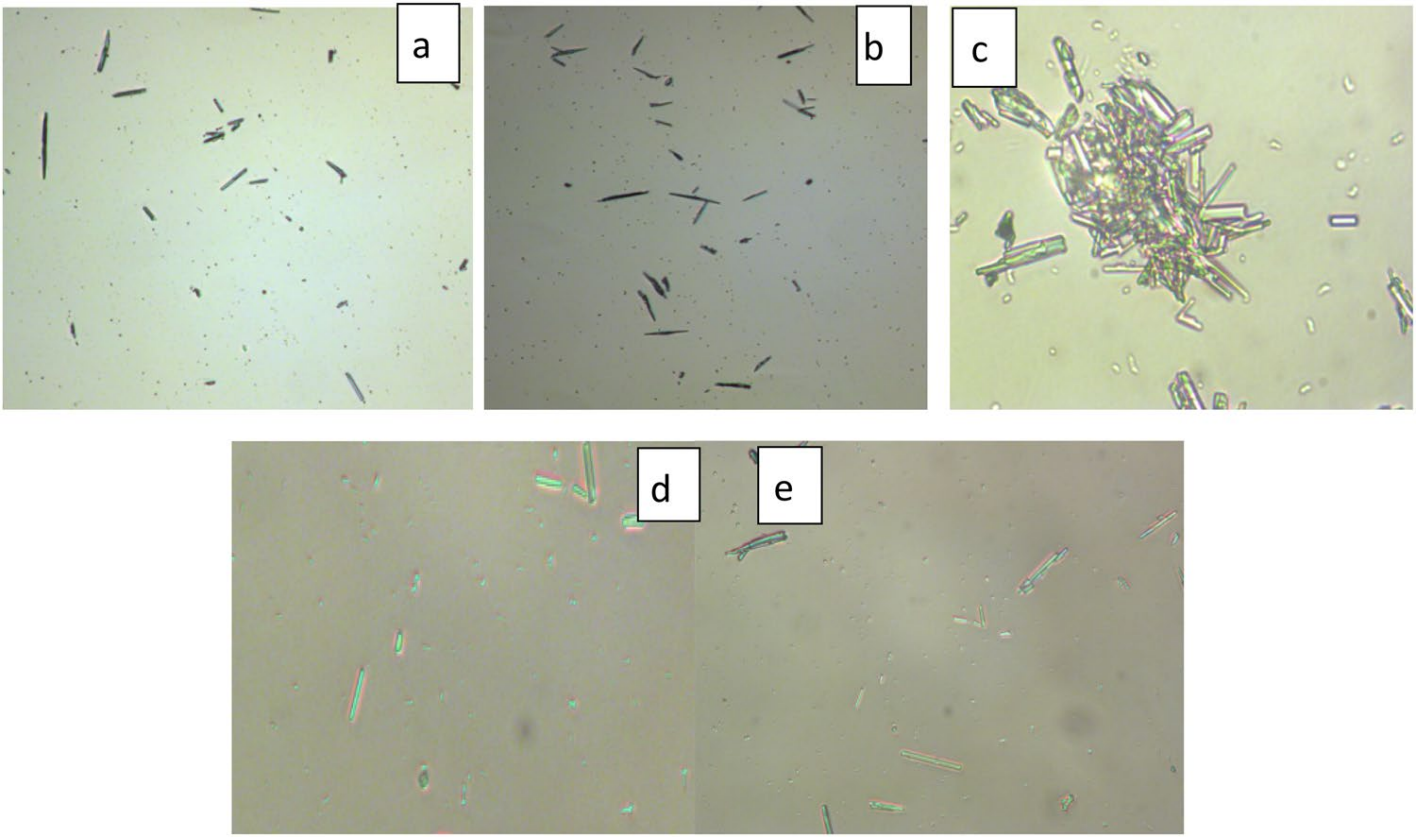
Microscope pictures of (**a**) pure oil and oil that had been treated with (**b**) CP_1_, (**c**) CP_2_, (**d**) NP_1_, and (**e**) NP_2_ at 0 °C.

With the use of a polarized optical microscope (POM), it was possible to trace how temperature affects morphology, size and dispersion of lubricating wax crystals^53^. Figure 12 displays microscope pictures of pure oil and oil that had been treated with CP_1_, CP_2_, NP_1_, and NP_2_ at 0 °C. As seen in Fig. 12a, the pure lubricating oil wax crystals has larger size, which resembled like disorganized, and they increased quickly as the temperature dropped. Thus lubricating fluid lost its capacity to flow because they were layered on top of one another and interfered to form 3D network. Figure 12b,c, respectively, illustrates the shape of wax crystals formed in oil after being treated by CP_1_ and CP_2_ at low temperatures where wax crystals are obviously modified in terms of quantity, shape, and size. In comparison to pure oil, the size and quantity of wax crystals were decreased. At low temperatures, the lubricating oil treated with NP_1_ and NP_2_ Fig. 12d,e displayed the most noticeable effect. The wax crystals that did precipitate are very few and thus oil has no difficulty in dissolving wax crystals^53^.

### Comparison between Mobil DTE Oil and the mineral oil treated with NP_2_

The characteristic of the commercial Mobil DTE Oil is compared to the mineral oil that treated with NP_2_ (that give the best results) and illustrated in Table 4. The data reveals that the mineral oil that mixed with the synthesized NP_2_ show best results at PPT, kinematic viscosity and VI, to enhance the mineral oil that treated with the prepared polymers.

**Table 4:** Comparison between Mobil DTE oil and treated oil with NP_2_.

Property	Mobil DTE oil	Oil treated with NP2
PPT, °C, ASTM D97	− 15	− 33
Kinematic viscosity at 100 °C	10.9	8.381
Kinematic viscosity at 40 °C	95.1	56.18
Viscosity index, ASTM D2270	92	93.103

## Conclusions

In the present work octadecyl methacrylate copolymers with α-olefins (HD or DD) were prepared by solution polymerization and their nanocomposites were prepared by emulsion polymerization. The prepared additives are clarified by FTIR, ^1^HNMR, DLS, TEM and thermal analysis (TGA and DSC) that give high thermal stability. Polymers nancomposites show high resolution figures of TEM and DLS to prove their synthesis in nano scale. The prepared polymers and their nanocomposite are effective pour point reducers and cold flow enhancers for lube oil because they are dissolved well in the oil. A comparison between copolymers and their nanocomposites showed that the latter gave better results as PPD, FI and VM, where NP_2_ that has the best efficiency gave −33 °C and 93.103 for PPD and VI, respectively. According to POM data, shape and size of wax in blank lubricating oil has changed upon treating with additives where it becomes tinier and separated. At the end of the comparison between the lube oil treated with NP_2_ with that products at the market (Mobil DTE); it was found that the first one show higher efficiency with lower cost.

## Data Availability

The datasets used and/or analyzed during the current study are available from the corresponding author A.A. El-Segaey.
